# The Effect of Lymphovascular Invasion on Short-Term Tumor Recurrence and Progression in Stage T1 Bladder Cancer

**DOI:** 10.7759/cureus.54844

**Published:** 2024-02-24

**Authors:** Osman Gercek, Melih Senkol, Veli Mert Yazar, Kutay Topal

**Affiliations:** 1 Urology, Afyonkarahisar Health Sciences University, Afyonkarahisar, TUR; 2 Urology, Afyonkarahisar State Hospital, Afyonkarahisar, TUR

**Keywords:** malignant tumor resection, lymphatic metastasis, disease progression, recurrence, bladder cancer

## Abstract

Introduction

Lymphovascular invasion (LVI) is the most important stage for tumor spread and metastasis. The role of LVI in transurethral resection is not yet clear. In this study, the progression and recurrences of patients who underwent transurethral resection bladder tumor (TUR-BT) and T1 high-grade tumor and concomitant LVI were detected in pathology results and were evaluated.

Methods

Our study included 58 patients, who underwent TUR-BT with the suspicion of bladder cancer and were pathologically diagnosed with T1 stage bladder cancer and who did not undergo radical surgery, in the Urology Clinic of Afyonkarahisar Health Sciences University, Turkey. The patient’s age, gender, tumor size, tumor grade, presence of LVI, second resection, recurrence, and progression rates at three months and one year were compared.

Results

LVI was detected in the pathology specimens of nine (15.5%) of the 58 patients who were included in the study. When the one-year progression was evaluated, progression to T2 tumor was detected in six (66.7%) patients in the group with LVI and five (10.2%) patients in the group without LVI, and the progression was significantly higher in the group with LVI (p=0.001). In logistic regression analysis, the only significant predictor for one-year progression was the presence of LVI (p=0.001).

Conclusion

According to the results of our study, the presence of LVI in the pathology specimens of patients with T1 high grade significantly increases the progression. Suggesting radical cystectomy and neoadjuvant chemotherapy to patients with LVI in the early period seems to be a more accurate approach, considering the course of the disease.

## Introduction

Bladder cancer is the fourth most common cancer diagnosed in men worldwide [1]. In Western countries, 70% of patients have non-muscle invasive bladder cancer (NMIBC) at the time of diagnosis [2]. Transurethral resection of bladder tumor (TUR-BT) is required to diagnose bladder cancer and make risk classification. After TUR-BT and intravesical bacillus Calmette-Guerin (BCG) treatment, 17% of the patients progress to muscle-invasive bladder cancer (MIBC) within 1 year [2,3].

The disease progresses in 20% of patients with T1 high-grade bladder cancer who undergo adequate TUR-BT and subsequently receive BCG [3,4]. The survival of these patients after radical cystectomy is longer than patients with MIBC at the time of diagnosis [5]. Recognizing the high-risk patients and directing them to radical cystectomy provides benefits both in terms of disease-related survival and reducing the overtreatment rates in BCG therapy given to patients. Prognosis models are associated with histopathological types of the disease, age, gender, clinical stage, recurrence rates, concomitant carcinoma in situ, tumor size, and tumor number [2,3,6]. The response to BCG therapy is lower in patients with variant histological subtypes of bladder cancer. Progression is faster in these patients [7,8]. Lymphovascular invasion (LVI) is a characteristic pathological finding in terms of progression. It especially plays a role in the cancer-specific survival of T1 high-grade patients [9-11].

LVI is the most important stage for tumor spread and metastasis [12]. The presence of LVI in patients treated with radical cystectomy indicates that the disease has an aggressive course. The role of LVI in transurethral resection is not yet clear [13-15]. In a meta-analysis, it was observed that patients with LVI in TUR-BT pathology had higher pathological stages and a poor clinical course [16].

In this study, the progression and recurrences of patients who underwent TUR-BT and who had T1 high-grade tumors and LVI were evaluated.

## Materials and methods

This study was conducted in the Urology Clinic of Afyonkarahisar Health Sciences University, Faculty of Medicine, Turkey. We tried to reach all patients between the specified dates. The data were retrospectively recorded after obtaining ethical approval (Afyonkarahisar Health Sciences University Clinical Research Ethics Committee, 2011-KAEK-2, 2023/327). The study was conducted per the principles of the Declaration of Helsinki.

A total of 81 patients were included in the study, who underwent TUR-BT with the suspicion of bladder cancer in the Urology Clinic of Afyonkarahisar Health Sciences University Faculty of Medicine between May 2016 and May 2022 and were found to have T1 stage bladder cancer pathologically. All patients included were classified as high and very high risk per European Association of Urology (EAU) guidelines. All patients with LVI were in the very high-risk category [17]. A second resection was planned for all patients four to six weeks later. Intravesical BCG therapy was recommended for three years to all patients who were in the high-risk group and had no contraindications. Radical cystectomy was recommended to patients who were in the very high-risk class and who did not respond to BCG, because of the high risk of recurrence and progression [18].

Eleven patients who did not attend their regular follow-ups for at least one year and who did not accept or comply with the recommended intravesical BCG therapy were excluded from the study. Radical cystectomy was performed on 12 patients with very high-risk bladder cancer or who were unresponsive to BCG and accepted radical surgery. Finally, the study continued with 58 patients with T1 stage bladder cancer, who attended their regular follow-up visits for at least one year, and who complied with intravesical BCG therapy, including the ones who were not appropriate for radical surgery because of their comorbidities or did not want the operation. The data including the patient’s age, gender, tumor size, tumor grade (low grade and high grade), presence of LVI, second resection, recurrence, and progression at the third and twelfth months were scanned and recorded. Progression was considered as progression to stage T2 disease. Tumor size was calculated as the longest diameter of the present tumor. Tumor size was measured using the loop width as a reference. All operations were performed by surgeons with similar experience.

Statistical analysis

Statistical analysis of the study data was performed with the Statistical Package for the Social Sciences (version 20.0; IBM SPSS Statistics for Windows, Armonk, NY). The conformity of the variables to the normal distribution was examined using the Kolmogorov-Smirnov (K-S) test. In the comparison of binary groups, the Student's t-test was used for normally distributed parameters, and the Mann-Whitney U test was used for non-normally distributed parameters. Evaluation of multi-well crosstabs was performed with the chi-square test or Fisher’s exact test. Logistic regression analysis was used to determine the factors affecting one-year recurrence and progression. Results were considered statistically significant when p<0.05.

## Results

LVI was detected in the pathology specimens of nine (15.5%) of the 58 patients included in the study. The mean age of all patients was 66.78±9.78 years, and no significant difference was found between the groups (p=0.103). When tumor grades were evaluated, low-grade tumors were detected in two (22.2%) patients in the group with LVI and in 16 (32.6%) patients in the group without LVI, and there was no statistical difference between the groups (p=0.706). The mean tumor size of both two groups was 49.52±28.40 mm, and there was no significant difference between the groups regarding the size (p=0.114) (Table 1).

**Table 1:** Demographic and clinical data of the patients *: Chi-square, **: Progression was detected in two patients during the second resection, LVI: Lymphovascular invasion, SD: Standard deviation

Demographic and clinical data	LVI Yes	LVI No	P value
	N=9	N=49	
Age (years, mean±SD)	71.67±8.91	65.88±9.74	0.103
Gender (n %)			
Male	8 (88.8)	47 (95.9)	0.403*
Female	1 (11.2)	2 (4.1)	
Tumor grade (n %)			
Low grade	2 (22.2)	16 (32.6)	0.706*
High grade	7 (77.8)	33 (67.4)	
Tumor size (mm, mean±SD)	60.00±24.66	47.59±28.84	0.114
Second resection T2 tumor (n %)			
Yes	2 (22.2)	0 (0)	0.022*
No	7 (77.8)	49 (100)	
One-year recurrence (n %)			
Yes	9 (100)	43 (87.8)	0.576*
No	0 (0)	6 (12.2)	
Except for the second resection one-year recurrence (n %)			
Yes	8 (88.9)	28 (57.1)	0.133*
No	1 (11.1)	21 (42.9)	
Except for the second resection one-year progression (n %)			
Yes	4 (44.4)	5 (10.2)	0.025*
No	5 (55.6)**	44 (89.8)	

A second resection was performed in all of the 58 patients included in the study. When the patients with T2 tumors in the pathology of the second resection were evaluated, while T2 tumors were detected in two (22.2%) patients in the group with LVI, there were no T2 tumors in patients in the group without LVI. Cystectomy was recommended again for two patients in whom T2 tumors were detected in the second resection. A cystectomy operation was performed on one patient. As the other patient did not want surgery, multimodal treatment was planned. The number of patients with T2 tumors at the second resection was significantly higher in the group with LVI (p=0.022). When one-year recurrence was evaluated, recurrence was found in all patients in the LVI group, but no statistically significant difference was found between the groups (p=0.576). Moreover, no significant difference was found in terms of recurrence between the groups, when classification was made excluding the second resection (p=0.133). When one-year progression was evaluated (two patients with T2 tumor at second resection were excluded), progression to T2 tumor was detected in four (66.7%) patients in the group with LVI and in five (10.2%) patients in the group without LVI, and the progression was significantly higher in the group with LVI (p=0.025) (Table 1).

The third-month cystoscopy findings have an important place in the follow-up of bladder cancer and the prediction of prognosis. When the third-month findings of the groups were evaluated separately, there was no difference between the groups in terms of recurrence rates, but statistically significantly higher progression rates were observed in the group with LVI (p=0.122, p=0.011, respectively) (Table 2).

**Table 2:** Comparison of the recurrence and progression of the groups in the third month *: Chi-square, LVI: Lymphovascular invasion

Third-month recurrence and progression	LVI Yes	LVI No	P value
	N=7	N=49	
	n %	n %	
Third-month recurrence			
Yes	5 (71.4)	18 (38.3)	0.122*
No	2 (28.6)	29 (61.7)	
Third-month progression			
Yes	3 (42.9)	2 (4.1)	0.011*
No	4 (57.1)	47 (95.9)	

Binary logistic regression analysis was performed to determine the possible independent predictors of one-year recurrence and progression that contributed the most to the outcome. Gender, age, tumor size, tumor grade, and presence of LVI were used as predictors. The model that predicted the one-year progression was significant (χ2(8)=5.9, p=0.652) and could explain 45.4% of the variance in reincarceration (Nagelkerke R2=0.454). The model correctly predicted that 95.7% of non-recurred and 54.5% of recurred patients (87.9% in total). The only significant predictor for one-year progression was the presence of LVI (p=0.001). The presence of LVI increased the risk of progression 3.54 times. In the model predicting one-year recurrence, no predictors were found to have a significant effect on recurrence (Table 3).

**Table 3:** Logistic regression of the predictors for one-year recurrence and progression Logistic regression analysis was used to arrive at the P value. A P value<0.05 is considered statistically significant. LVI: Lymphovascular invasion, RR: relative risk reported with odds ratio, CI: confidence interval

Risk factors	Progression		Recurrence	
	RR (%95 CI)	P value	RR (%95 CI)	P value
Age (years)	0.973 (0.877-1.080)	0.611	1.029 (0.965-1.096)	0.384
Gender	0.000 (0.000-0.000)	0.999	0.000 (0.000-0.000)	0.999
Tumor grade	0.256 (0.033-1.975)	0.191	1.948 (0.508-7.468)	0.331
Tumor size	1.025 (0.998-1.054)	0.070	1.000 (0.979-1.023)	0.966
Presence of LVI	34.574 (4.093-292.031)	0.001	4.562 (0.500-41.604)	0.178

## Discussion

The mechanism of lymphatic metastasis is still not yet clearly defined. It probably starts with the migration of cancer cells to the lymphovascular area at a microscopic level before the development of fulminant lymph node metastasis [19]. LVI indicates that tumor invasion has reached the lymphatic and vascular parts. It may also be a sign that cancer cells have passed into the metastatic phase [20]. LVI is an important step for the systemic spread of cancer cells [21]. Studies performed have shown that LVI is an independent risk factor for the recurrence of the disease [22,23]. However, most of these studies have been studied pathologically after cystectomy. Similar risk factors are also valid in cancers originating from the urothelium of the upper urinary system [14,24].

In the study by D’Andrea et al., wherein 1,289 patients were followed for a mean of 56 months, disease progression was observed to be statistically significantly increased in patients with variant histology and LVI. While progression and recurrence increased in patients with variant histology, progression was significantly increased in patients with LVI, but there was no significant increase in recurrence [25]. In our study, a significant increase in progression was observed in patients with LVI. In our study, we found a nominal increase in the number of recurrences, but it was not statistically significant.

There are limited studies on the effect of LVI on disease-related recurrence in patients with T1 high-grade tumors. In the study conducted by Cho et al., it has been shown that, in newly diagnosed T1 high-grade patients, LVI increases the risk of disease progression and metastasis [26]. Moreover, as a result of our study, LVI increased the progression. In these patients, early cystectomy and possibly neoadjuvant chemotherapy are beneficial. Multiple intravesical treatments are considered ineffective in these patients [27].

There are some limitations in detecting lymphovascular invasion after TUR-BT. One of them is false positivity or false negativity. Retraction artifacts may mimic LVI pathologically [28]. Additionally, the diagnosis of LVI needs to be standardized. Immunohistochemical methods such as CD31 and CD34 can be used [29].

Although studies have shown a significant association between tumor size and tumor grade with stage progression, the present study did not show any such association [30].

We acknowledge the limitations of our study. The major limitation of the study is that this is a retrospective study. The other limitations of our study are the low number of patients because of the homogeneous patient group and the inability to evaluate the genetic, tumor-specific, and other factors affecting recurrence, and progression.

## Conclusions

In our study findings, the presence of LVI in the pathology specimens of patients with T1 high grade significantly increases the progression. In our study, factors affecting progression were investigated, and it was found that the presence of LVI affected progression more than tumor size and grade. We think that patients with LVI should be offered radical cystectomy and neoadjuvant chemotherapy in the early period, and time should not be wasted considering the failure of intravesical therapy. Multicenter prospective studies with a higher number of patients may highlight the importance of LVI in bladder cancer more significantly and in detail.
